# Ruptured Bladder

**Published:** 1894-01

**Authors:** Leon N. Reefer


### RUPTURED BLADDER.

By Leon N. Reefer, V.M.D.

On the 21st of February, 1893, I was called to attend an ox, the property of Wm. Dixon, at Rosby's Rock, Marshall Co., West Virginia. I found a fine big animal weighing about 1900 and in apparently good condition, but very weak; he had the dejected look of an animal suffering from some chronic trouble, but with no acute symptoms whatever.

Upon inquiry I found that the beast had been suffering for twenty-two days and that in that period he had only urinated freely but once, and this time was after he had been suffering great pain for two days, immediately after being taken with his sickness. He had eaten somewhat up to the time I had been called, but now was standing in a very tottering manner, and apparently in a dying condition. My diagnosis was, that the animal was suffering with urinary trouble, but with such a history I was dumbfounded. On rectal examination of the bladder I found it in a flabby condition, containing no urine, and a rupture in its walls. During this examination the presence of fluid in the abdominal cavity was readily detected, and the presence of this is what accounted for his fine appearance, as it filled him out and made him look in very good condition. While we were at our supper the animal died, and I immediately proposed a post-mortem examination. After removing the skin and placing him on his back, I made an opening near the edges of the lumbar spinous processes, and by means of a two-gallon bucket I was enabled to measure the contents of this cavity. It contained seventy-two gallons of a clear transparent liquid with the decided odor of urine, and this quantity would be somewhat increased had we the opportunity of collecting it in total. The bladder was in its normal position, but its walls were shrivelled up and a rupture over two inches in length was found in the inferior surface. The lining membrane was thickened and nodulated, and the edges of the wound were indurated. The ruptuıe had been caused by a calculi lodging in the urethral canal, thus closing the passage of the urinary duct. All other organs were in a healthy condition, only somewhat anæmic. With the presence of this great amount of urine in this cavity for such a length of time, and no appearance of uraemic poisoning nor peritonitis, is very peculiar and without parallel in bovine medicine and surgery.

